# The Developing Human Connectome Project Neonatal Data Release

**DOI:** 10.3389/fnins.2022.886772

**Published:** 2022-05-23

**Authors:** A. David Edwards, Daniel Rueckert, Stephen M. Smith, Samy Abo Seada, Amir Alansary, Jennifer Almalbis, Joanna Allsop, Jesper Andersson, Tomoki Arichi, Sophie Arulkumaran, Matteo Bastiani, Dafnis Batalle, Luke Baxter, Jelena Bozek, Eleanor Braithwaite, Jacqueline Brandon, Olivia Carney, Andrew Chew, Daan Christiaens, Raymond Chung, Kathleen Colford, Lucilio Cordero-Grande, Serena J. Counsell, Harriet Cullen, John Cupitt, Charles Curtis, Alice Davidson, Maria Deprez, Louise Dillon, Konstantina Dimitrakopoulou, Ralica Dimitrova, Eugene Duff, Shona Falconer, Seyedeh-Rezvan Farahibozorg, Sean P. Fitzgibbon, Jianliang Gao, Andreia Gaspar, Nicholas Harper, Sam J. Harrison, Emer J. Hughes, Jana Hutter, Mark Jenkinson, Saad Jbabdi, Emily Jones, Vyacheslav Karolis, Vanessa Kyriakopoulou, Gregor Lenz, Antonios Makropoulos, Shaihan Malik, Luke Mason, Filippo Mortari, Chiara Nosarti, Rita G. Nunes, Camilla O’Keeffe, Jonathan O’Muircheartaigh, Hamel Patel, Jonathan Passerat-Palmbach, Maximillian Pietsch, Anthony N. Price, Emma C. Robinson, Mary A. Rutherford, Andreas Schuh, Stamatios Sotiropoulos, Johannes Steinweg, Rui Pedro Azeredo Gomes Teixeira, Tencho Tenev, Jacques-Donald Tournier, Nora Tusor, Alena Uus, Katy Vecchiato, Logan Z. J. Williams, Robert Wright, Julia Wurie, Joseph V. Hajnal

**Affiliations:** ^1^Centre for the Developing Brain, School of Biomedical Engineering and Imaging Sciences, King’s College London, London, United Kingdom; ^2^MRC Centre for Neurodevelopmental Disorders, King’s College London, London, United Kingdom; ^3^Biomedical Image Analysis Group, Department of Computing, Imperial College London, London, United Kingdom; ^4^Institute for AI and Informatics in Medicine, Klinikum Rechts der Isar, Technical University of Munich, Munich, Germany; ^5^Wellcome Centre for Integrative Neuroimaging, FMRIB, Nuffield Department of Clinical Neurosciences, University of Oxford, Oxford, United Kingdom; ^6^Biomedical Engineering Department, School of Biomedical Engineering & Imaging Sciences, King’s College London, London, United Kingdom; ^7^Sir Peter Mansfield Imaging Centre, Mental Health and Clinical Neurosciences, School of Medicine, University of Nottingham, Nottingham, United Kingdom; ^8^Department of Forensic and Neurodevelopmental Sciences, Institute of Psychiatry, Psychology & Neuroscience, King’s College London, London, United Kingdom; ^9^Faculty of Electrical Engineering and Computing, University of Zagreb, Zagreb, Croatia; ^10^Centre for Brain and Cognitive Development, Department of Psychological Sciences, Birkbeck, University of London, London, United Kingdom; ^11^Department of Electrical Engineering, ESAT/PSI, KU Leuven, Leuven, Belgium; ^12^BioResource Centre, NIHR Biomedical Research Centre, South London and Maudsley NHS Trust, London, United Kingdom; ^13^Biomedical Image Technologies, ETSI Telecomunicación, Universidad Politécnica de Madrid and CIBER-BBN, Madrid, Spain; ^14^Department of Medical and Molecular Genetics, School of Basic and Medical Biosciences, King’s College London, London, United Kingdom; ^15^Translational Bioinformatics Platform, NIHR Biomedical Research Centre, Guy’s and St. Thomas’ NHS Foundation Trust and King’s College London, London, United Kingdom; ^16^Institute for Systems and Robotics (ISR-Lisboa)/LaRSyS, Department of Bioengineering, Instituto Superior Técnico, Universidade de Lisboa, Lisbon, Portugal; ^17^Department of Child and Adolescent Psychiatry, Institute of Psychiatry, Psychology and Neuroscience, King’s College London, London, United Kingdom

**Keywords:** Developing Human Connectome Project, brain development, MRI, neonatal, connectome, perinatal

## Abstract

The Developing Human Connectome Project has created a large open science resource which provides researchers with data for investigating typical and atypical brain development across the perinatal period. It has collected 1228 multimodal magnetic resonance imaging (MRI) brain datasets from 1173 fetal and/or neonatal participants, together with collateral demographic, clinical, family, neurocognitive and genomic data from 1173 participants, together with collateral demographic, clinical, family, neurocognitive and genomic data. All subjects were studied *in utero* and/or soon after birth on a single MRI scanner using specially developed scanning sequences which included novel motion-tolerant imaging methods. Imaging data are complemented by rich demographic, clinical, neurodevelopmental, and genomic information. The project is now releasing a large set of neonatal data; fetal data will be described and released separately. This release includes scans from 783 infants of whom: 583 were healthy infants born at term; as well as preterm infants; and infants at high risk of atypical neurocognitive development. Many infants were imaged more than once to provide longitudinal data, and the total number of datasets being released is 887. We now describe the dHCP image acquisition and processing protocols, summarize the available imaging and collateral data, and provide information on how the data can be accessed.

## Introduction

Recent advances in MRI acquisition, image processing and analysis have made it possible to gain a non-invasive yet detailed multimodal characterization of the human brain’s macroscopic connections (Craddock et al., 2013). Novel connectivity maps encompass not only the structural connections relating to white matter tracts, but the functional connections revealed by coordinated gray-matter activations, and connectivity related to coordinated development revealed in structural covariance (Alexander-Bloch et al., 2013) and multimodal similarity networks (Seidlitz et al., 2018). The value of these approaches has been highlighted in recent years by the Human Connectome Project (HCP), which has fostered growing interest in the science of connectomics and become a critical resource for research into the mature human brain (Van Essen et al., 2013).

Human brain development accelerates rapidly in late pregnancy to reach maximum global growth rate before 6 months (Bethlehem et al., 2022). This rapid growth is accompanied by equally dramatic changes in the brain’s associated architecture of structural and functional connectivity, and therefore understanding these processes in both the healthy and pathological brain can provide marked new insights into fundamental neural processes and the possible changes that underlie intractable neuropsychiatric conditions. However, characterization of this process has previously been limited by the challenges inherent in safely and robustly studying the brain during this vulnerable phase of life. The Developing Human Connectome Project (dHCP) is an open science study, funded by the European Research Council to obtain and disseminate Magnetic Resonance Imaging (MRI) data which map the brain’s structural and functional development across the period from 20 weeks gestational age to full term. By coupling advances in imaging with bespoke solutions developed for the fetal and neonatal population, principally but not exclusively solving the problems of subject motion, the dHCP captures the development of brain anatomy and connectivity at a systems level. This enables exploration of maturational trajectories, structure and function relationships, the neural substrates for behavior and cognition, and the influences of genetic and environmental factors. The dHCP includes both *in utero* imaging of fetal brain and postnatal imaging of preterm and term born infants, capturing typical and atypical brain development. It has created maps of the developing human brain and its connections as a resource for the neuroscience community and a platform for connectome research.

The dHCP dataset includes a large number of healthy, term-born infants which allow definition of typical development with previously unobtainable precision. It is increasingly appreciated that the perinatal period is crucial for lifelong brain health, and multiple lines of evidence show that early life influences have a critical effect on brain circuitry in later childhood and adult life (Batalle et al., 2018). This has key implications for understanding the pathophysiology of neurodevelopmental conditions, such as autism (Hisle-Gorman et al., 2018) or the difficulties associated with preterm birth (Montagna and Nosarti, 2016). Understanding these effects has important clinical implications, and to support relevant investigations clinical and demographic data were collected and saliva samples obtained for genetic and epigenetic analysis, with participating families invited back at 18 months of age for a developmental assessment using standard tests and questionnaires, including eye-tracking studies.

A key priority for the project was that the data be made available to the research community, and preliminary data releases^1^ have been accessed and used by a number of research groups. We now describe the main neonatal data release, providing a summary of the participants, the MR imaging data acquisition and processing, the collateral data including sociodemographic and neuropsychological outcome data, and the genomic data. We also describe available data for each category and how to obtain it. Fetal data will be described and released separately.

## Participants

Infants were recruited at St Thomas’ Hospital, London and imaged at the Evelina Newborn Imaging Centre, Centre for the Developing Brain, King’s College London, United Kingdom. The MR suite is sited within the neonatal intensive care unit which allows imaging of even the smallest and most vulnerable newborn infants, as well as having proximity to the maternity unit to support fetal scanning.

**FIGURE 1:**
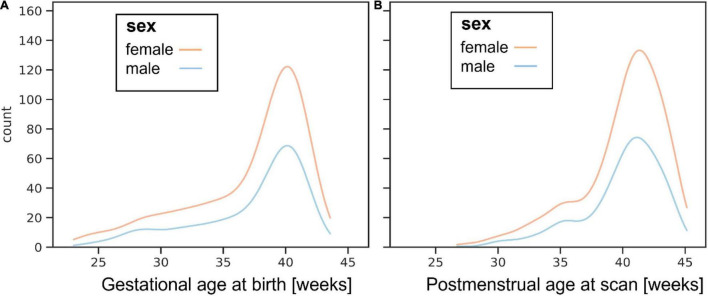
Histograms showing ages for boys and girls at **(A)** birth and **(B)** postnatal MR imaging.

The images of 783 newborn infants are being released. Infants were recruited with specified inclusion and exclusion criteria^2^ across a spread of gestational ages at birth (range: 23 to 43 + 1 weeks + days) and post-menstrual ages at the time of study (range: 26 + 5 to 45 + 1). The distributions of gestational age at birth and post-menstrual age at scan are shown in Figure 1.

The study population includes 583 subjects born at term equivalent age (37–44 weeks post-menstrual age) without any known pregnancy or neonatal problems and are regarded as healthy. All the anatomical images were reviewed by an expert perinatal neuroradiologist and radiologic scores included in the released data. Incidental findings were noted in a proportion and a report on these have been published (Carney et al., 2021).

## Magnetic Resonance Imaging Data

### Overview

**FIGURE 2:**
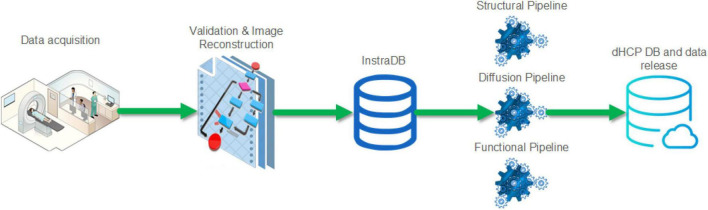
Schematic of the Developing Human Connectome Project imaging data flow from acquisition to data release.

A summary schematic of the imaging data flow is shown in Figure 2 with further detail about the steps in the following section. This incorporated optimized MR acquisition sequences, novel image reconstruction methods, transfer to an intermediate server (InstraDB) prior to processing using state-of-the-art pipelines, and packaging of the data for final public release.

The data release contains anatomical [T1 weighted (T1w) and T2 weighted (T2w)], resting state functional MRI (rsfMRI) and diffusion MRI (dMRI) images supplied as both original image data and after the processing pipelines described below have been applied.

The neonatal brain has significantly different tissue properties to the adult brain, including higher water content and incomplete myelination of white matter, and T1 and T2 relaxation times are generally longer than in the adult brain. Neonatal white matter in particular, has longer T1 and T2 times in comparison to gray matter, and brain anatomy is revealed more clearly on T2w images as there is greater contrast compared to T1w images. T2w images are thus treated as the primary data for anatomical segmentation and to provide the anatomical substrates for functional and diffusion analysis.

To ameliorate the effects of infants and fetuses moving during image acquisition novel neonatal patient handling and motion-tolerant acquisition approaches were developed (Hughes et al., 2017; Cordero-Grande et al., 2018, 2020; Hutter et al., 2018a). Participants were imaged in natural sleep, with six exceptions who were sedated with chloral hydrate. If a baby woke up, scanning was halted and the infant settled without taking them out of the imaging cradle. However, as many infants still move even when sleeping peacefully, all subjects were motion corrected.

A total of 887 sessions are being released. 886 had T2w images that passed quality control (QC). 818 had fMRI data that passed QC and 758 had dMRI data that passed QC. Detailed information about the QC process are described in the notes accompanying the data release.^3^ The T1w images were not required by pre-processing pipelines and were placed at the end of the scanning protocol resulting in more variable quality than the T2w data; the release contains 711 sessions with T1w multi-slice fast spin-echo (FSE) images and 734 sessions with T1 3D magnetization-prepared rapid gradient-echo (MPRAGE) images.

### Imaging Acquisition Methods and Parameters

Imaging was carried out on a 3T Philips Achieva scanner running modified Release 3.2.2 software, using a dedicated neonatal imaging system which included a neonatal 32 channel phased array head coil and customized patient handling system (Rapid Biomedical GmbH, Rimpar, Germany) (Hughes et al., 2017). Infants were imaged following feeding and swaddling in a vacuum-evacuated blanket. Infants were provided with hearing protection in the form of: molded dental putty placed in the external auditory meatus (President Putty, Coltene Whaledent, Mahwah, NJ, United States); Minimuffs (Natus Medical Inc., San Carlos, CA, United States); and an acoustic hood. Monitoring throughout the scanning session (*In vivo* Expression, Philips, Best, NL), included pulse oximetry, respiration (using a small air cushion placed on the lower abdomen) and body temperature *via* a fiber optic probe placed in the axilla. The bespoke imaging cradle system (Hughes et al., 2017) placed subjects in a standardized pose and allowed a fixed imaging geometry to be deployed, with only the position in the head-foot direction adjusted at the start of the examination. The field of view was set after a biometric analysis of data from 91 previously studied term-born infants with dimensions sufficient to accommodate 95% of late-term neonates (Hughes et al., 2017).

To reduce the risk of waking infants due to startle responses at the start of new sequences, the scanner software was modified to ramp up the gradient waveforms gradually over 5 s as each acquisition commenced and prior to any radiofrequency (RF) pulses or data being acquired. Calibration scans, anatomical images (T1w and T2w), resting state functional (rs-fMRI) and diffusion (dMRI) acquisitions were acquired, with an average data rate of 27 slices/second including all preparation and calibration phases. The acquisition protocol was optimized for the properties of the neonatal brain and for efficiency and is summarized in Table 1.

**TABLE 1:** Neonatal imaging protocol, lasting a total of 1 h 3 min 11 s.

Sequence name	Duration	Acquisition reference publications	Processing pipeline reference publications
Pilot	00:00:10		
Coil reference	00:01:14		
B0 calibration map	00:00:20	Gaspar et al., 2015	
B1 map	00:00:05		
T2 Turbo Spin Echo (TSE) axial	00:03:12	Cordero-Grande et al., 2016; Hughes et al., 2017; Cordero-Grande et al., 2018	Schuh et al., 2017; Makropoulos et al., 2018
T1 MPRAGE	00:04:35		
T2 TSE sagittal	00:03:12		
Spin Echo (SE) fMRI ref.	00:01:53	Price et al., in preparation	Baxter et al., 2019; Fitzgibbon et al., 2020
Single-Band (SB) fMRI ref.	00:00:19		
Multi-Band (MB) fMRI	00:15:03		
SB fMRI ref. repeat	00:00:19		
SB diffusion MRI ref.	00:01:39	Cordero-Grande et al., 2018; Hutter et al., 2018a,b; Cordero-Grande et al., 2019; Tournier et al., 2020	Bastiani et al., 2019; Christiaens et al., 2019; Pietsch et al., 2019; Christiaens et al., 2021
MB diffusion MRI	00:19:20		
B0 shim map	00:00:20		
T1 TSE Inversion Recovery (IR) axial	00:05:45	Cordero-Grande et al., 2018	
T1 TSE IR sagittal	00:05:45		
Total	01:03:11		

#### Calibration Scans

Static magnetic field (B0) mapping was performed using an interleaved dual TE spoiled gradient echo sequence and localized image-based shimming performed for use with all EPI sequences (Gaspar et al., 2015). Following application of optimized 1st and 2nd order shim settings, B0 (shimmed) field maps were acquired after the fMRI and dMRI acquisitions, and later in the cohort were acquired between the two acquisitions. B1 mapping was performed using the dual refocusing echo acquisition mode (DREAM) method (Nehrke and Bornert, 2012), with STE first and STEAM flip angle of 60.

#### Anatomical Acquisition

Imaging parameters were optimized for contrast to noise ratio using a Cramer Rao Lower bound approach (Lankford and Does, 2013) with nominal relaxation parameter values for gray matter T1/T2: 1800/150 ms and white matter T1/T2: 2500/250 ms (Williams et al., 2005). T2w and inversion recovery T1w multi-slice FSE images were each acquired in sagittal and axial slice stacks with in-plane resolution 0.8 × 0.8 mm^2^ and 1.6 mm slices overlapped by 0.8 mm (except in T1w Sagittal which used a slice overlap of 0.74 mm). Other parameters were–T2w: TR/TE = 12000/156 ms, SENSE factor 2.11 (axial) and 2.60 (sagittal); T1w: TR/TI/TE = 4795/1740/8.7 ms, SENSE factor 2.27 (axial) and 2.66 (sagittal). 3D MPRAGE images were acquired with 0.8 mm isotropic resolution and parameters: TR/TI/TE = 11/1400/4.6 ms, SENSE factor 1.2 RL (Right-Left). The FSE acquisitions were each reconstructed using a motion correction algorithm and then the transverse and sagittal images were fused into a single 3D volume for each modality using slice-to-volume methods (Cordero-Grande et al., 2016).

#### Resting State Functional Magnetic Resonance Imaging

A fMRI acquisition with high temporal resolution developed for neonates (Price et al., in preparation; Fitzgibbon et al., 2020) using multiband (MB) 9× accelerated echo-planar imaging was collected for 15 min, with parameters: TE/TR = 38/392 ms, 2300 volumes, with an acquired spatial resolution of 2.15 mm isotropic. No in-plane acceleration or partial Fourier was used. Single-band reference scans were also acquired with bandwidth matched readout, along with additional spin-echo acquisitions with both anterior-posterior/posterior-anterior (AP/PA) fold-over encoding directions. Physiological recordings of vectorcardiogram (VCG), photoplethysmogram (PPU) and respiratory traces during the fMRI data acquisition are provided unprocessed in the source data folder for optional physiological artifact removal. Alignment to rs-fMRI data can be achieved by means of locating the “end of scan” marker (scripts are available to aid loading and interpretation of this file) and knowledge of the frequency of the recordings (496 Hz) and TR × number of volumes acquired (0.392 s × 2300) can be used to identify the start of scan timepoint. Note, for improved accuracy on this cohort a small delay of ∼85 ms between the true end of data acquisition and “end of scan” marker has been identified. After accounting for this, the precision of identifying the true start of scan in the physiological file should be on the order of ±50 ms, for a complete scan of 15 min duration.

#### Diffusion Magnetic Resonance Imaging

The dMRI acquisition was optimized for the properties of the developing brain (Tournier et al., 2020) and implemented as a uniformly distributed set of directions on 4 shells (*b* = 0 s/mm^2^: 20, *b* = 400 s/mm^2^: 64, *b* = 1000 s/mm^2^: 88, *b* = 2600 s/mm^2^: 128), each of which was split into 4 optimal subsets acquired using AP, PA, RL, and LR phase encoding (Hutter et al., 2018b). As described in Hutter et al. (2018b), the diffusion gradient *b*-values and directions and the phase encoding directions were spread temporally taking the risk of infant motion and gradient duty cycle considerations into account in order to achieve maximal imaging efficiency. If the subject woke up during the diffusion scan, the acquisition could be halted and restarted (after resettling the subject) with a user defined overlap in acquired diffusion weightings. The EPI sequence uses MB factor 4, SENSE factor 1.2, partial Fourier factor 0.86, in-plane resolution 1.5 × 1.5 mm, 3 mm slices with 1.5 mm overlap, TE = 90 ms, TR = 3800 ms. Image reconstruction used a dedicated SENSE algorithm (Hennel et al., 2016; Zhu et al., 2016; Cordero-Grande et al., 2018).

### Processing Pipelines

Standardized processing pipelines for all three MRI modalities (anatomical, diffusion, and functional imaging) have been developed specifically for the dHCP neonatal data. The outputs of these pipelines are supplied as part of the data release. Details of the individual pipelines have been published elsewhere including: anatomical segmentations into 9 tissues and 87 regions, and extracted cortical surfaces (Makropoulos et al., 2018) and cortical atlases (Bozek et al., 2018), resting state fMRI analysis (Fitzgibbon et al., 2020) and two diffusion analysis pipelines based on FSL EDDY (Bastiani et al., 2019) and based on SHARD slice-to-volume reconstruction (Christiaens et al., 2019, 2021). The SHARD pipeline also includes de-noised source diffusion data (Cordero-Grande et al., 2019) and inter-slice intensity correction (Pietsch et al., 2021). These offer natural entry points for those wishing to use image analysis software such as FSL^4^ and MRtrix3^5^ for further analysis. An atlas of diffusion properties has also been created based on a multi-shell multi-tissue constrained spherical deconvolution model (Pietsch et al., 2019). Whilst the majority of the processing pipelines are designed specifically for neonatal data given the inherent differences in tissue contrast and image properties, most analysis pipelines were also set up for comparison with adult data in mind. For instance, the cortical analysis pipeline was aligned with the young adult HCP FS_LR template space. However, we would urge caution about directly comparing adult and neonatal data given that much of the HCP dataset is aligned and parcellated using adult functional networks, and it is likely that the developing functional networks are not sufficiently developed to support this.

### Exemplar Imaging Data

**FIGURE 3:**
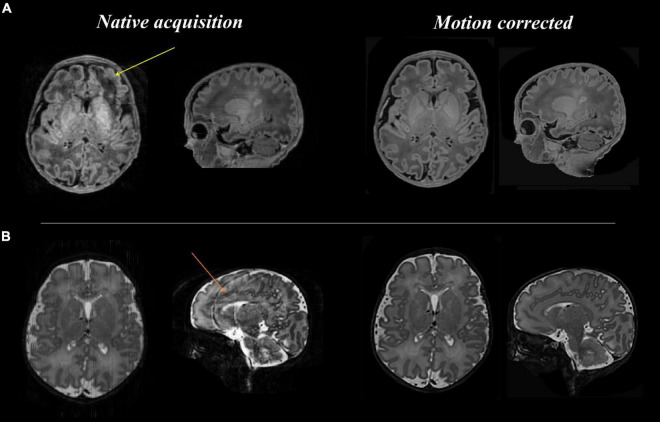
Anatomical T1 and T2 weighted images before and after motion correction for one participant. (**A:** top row) T1 native acquisition (left) with motion artifact visible in the left frontal region in the transverse plane (yellow arrow), which is resolved in the motion corrected images (right) after slice to volume reconstruction. (**B:** bottom row) T2 native acquisition (left) with motion artifact visible in the sagittal plane (orange arrow), which is resolved in the motion corrected images (right).

**FIGURE 4:**
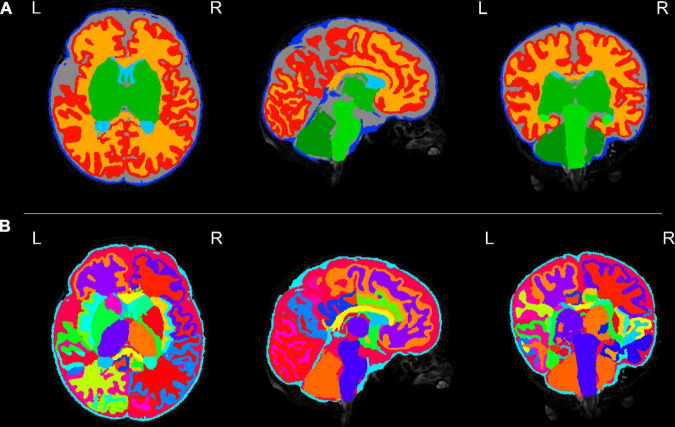
Tissue segmentation and neonatal atlas parcelation for the same infant. Using the automated dHCP structural pipeline, the anatomical images can be segmented into nine tissue classes (**A:** top row) and parcellated into 87 brain regions (**B:** bottom row).

**FIGURE 5:**
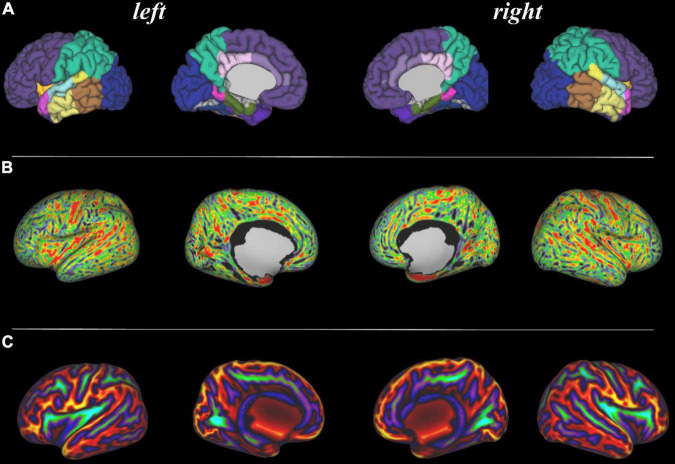
Surface projections using the dHCP structural pipeline for the same infant. (**A:** top row) 87 region neonatal brain atlas projected onto the pial surface; (**B:** middle row) Cortical thickness projected onto the inflated cortical surface; and (**C:** bottom row) Sulcal depth projected onto the inflated cortical surface.

**FIGURE 6:**
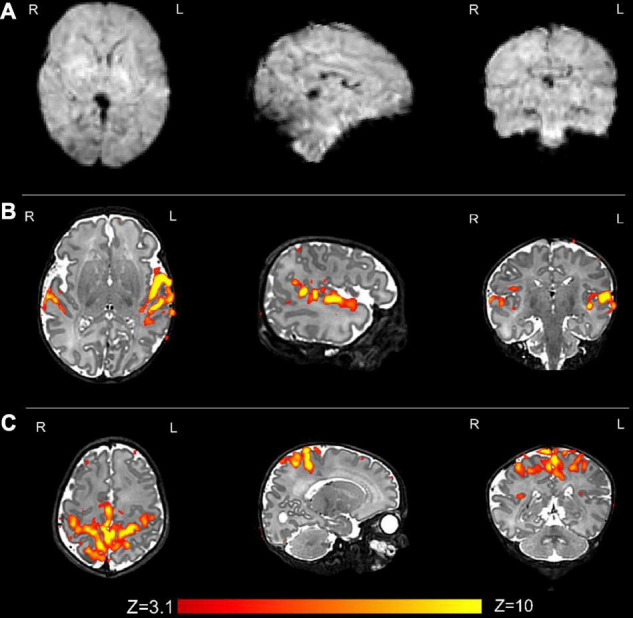
Resting state functional MRI data from the same infant. **(A)** An example volume from the fMRI acquisition after image reconstruction and the preprocessing pipeline has been applied; and **(B)** the auditory and **(C)** sensorimotor resting state networks. Resting state networks were defined using independent component analysis (ICA) as implemented in FSL MELODIC and have been overlaid onto the native T2 image for ease of visualization.

**FIGURE 7:**
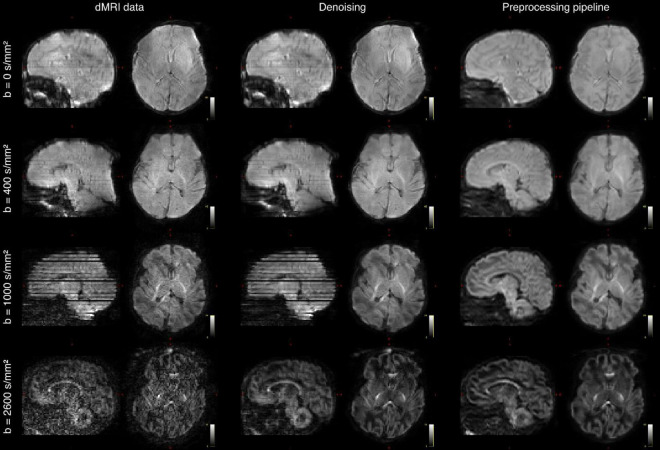
Diffusion MRI (dMRI) data from the same infant. Shown are four selected volumes with different *b*-values and phase encoding directions. Left: input data after MB-SENSE reconstruction. Middle: images after denoising. Right: images after motion and distortion correction and destriping.

**FIGURE 8:**
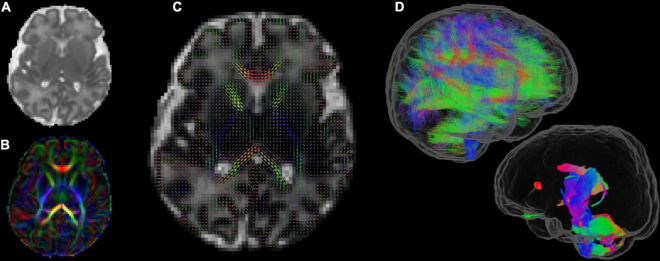
Diffusion MRI metrics in a single subject from the same infant **(A)** Mean Diffusivity and **(B)** Color Fractional Anisotropy maps of the Diffusion Tensor Imaging (DTI) model. **(C)** Tissue Orientation Distribution Function (ODF) of the multi-component analysis in Pietsch et al. (2019). **(D)** Full brain probabilistic streamline tractography based on the tissue ODF (top image) and based on the mature appearing tissue component (bottom image).

Figures 3–8 show examplar data for one participant to provide an indication of what is available. Figure 3 shows anatomical T1w and T2w fast spin echo data from this infant with the native images for all the acquisitions and the final motion corrected reconstructions. Although the infant was asleep, there is still some residual motion artifact. However, the final reconstruction can be seen to be of high quality after motion correction. The MPRAGE data (not shown) is not motion corrected, so is more vulnerable to subject motion. The anatomical segmentation into tissue type and neonatal brain atlas regions are shown in Figure 4, and cortical surfaces with projection of the atlas and example derived measures for this subject are shown in Figure 5. Anatomical atlases at one week intervals are available for download^6^ and will also be available from the NIMH database.^7^ Figure 6 shows one volume of the fMRI time series and a single subject network analysis from the pipeline. Figures 7, 8 show diffusion data. Figure 7 shows selected images from all shells, before correction, after denoising, and after motion and distortion correction and destriping (Pietsch et al., 2021). Figure 8 shows derived dMRI metrics in the same slice, including the mean diffusivity (8a) and fractional anisotropy (8b) of the diffusion tensor (Basser et al., 1994), fiber orientation distribution functions (8c) estimated using multi-component spherical deconvolution (Jeurissen et al., 2014; Pietsch et al., 2019) produced with MRtrix3 (Tournier et al., 2019), and (9d) whole brain probabilistic streamline tractography using all tissue components and using only the mature white matter like component from the neonatal multi-component model (Pietsch et al., 2019).

## Collateral Data

A broad spread of demographic and other data is available, although practical constraints, including the COVID-19 pandemic, have led to a certain amount of missing data. The data codebook can currently be accessed through the dHCP website (see text footnote 1) and NIMH database (see text footnote 7), providing a listing with descriptions of the variables. The data sets include the following categories of data.

### Demographic, Family, and Clinical Data

#### Demographic Data for Parents

Age at conception; ethnicity according to United Kingdom census categories; highest age enrolled in full-time education; occupation. This data is collected at enrollment and again at the 18-month neurodevelopmental assessment.

#### Mother’s Past Medical History

Height, weight, body mass index (BMI); blood group; history of medical conditions prior to the pregnancy; smoking, alcohol, and recreational drug use; injury during the pregnancy.

#### Mother’s Obstetric History

Previous pregnancies; number of live births; number of miscarriages; previous premature birth; current pregnancy type, mode of conception (natural or IVF); pregnancy number; late pregnancy and labor/delivery history for the pregnancy.

#### Mental Health History

Self-reported by mother at enrollment and self-reported by both parents at the 18-month assessment, including any history of parental psychiatric problems and how treated; parental history of attention deficit hyperactivity disorder (ADHD), bipolar disease, autistic spectrum disorder (ASD), or schizophrenia; ASD or ADHD in proband’s siblings; close relatives with history of ASD, ADHD, bipolar disease, or schizophrenia.

#### Baby Medical Details at Birth

Gestational age at birth; birth weight, length, and occipito-frontal head circumference; presentation and mode of delivery; medication required at delivery, nutrition and feeding; Apgar scores at 1 and 5 min of age; arterial cord blood pH and base excess where available. The majority of dHCP participants were born in good health and were not admitted to the neonatal intensive care unit (NICU), for those who were, summary data for each day on the neonatal unit and an overall summary of the stay are recorded.

### Neurodevelopmental and Neurocognitive Testing at 18 Months

A series of standardized age-appropriate child-centered assessments, parent-report questionnaires, and gaze-tracking tasks were used to provide a targeted overview of toddlers’ development. These measures were chosen were chosen to be able to capture individual differences along a typical-to-atypical continuum, to probe associations between early imaging features and emerging behavioral outcomes and to provide normative reference data for future studies.

A total of 619 infants (79%) attended for follow-up assessment, planned for 18 months corrected age but affected by the COVID-19 pandemic, so that median (range) of assessment was 18 months + 12 days (range 17 + 8–34 + 15). Completion rates for broad components of this assessment are shown in Tables 2, 3.

**TABLE 2:** Completion rates for neurodevelopmental assessments and questionnaires.

Neurodevelopmental assessment/Questionnaire	Number (%)
Bayley III Cognitive, language, motor neurodevelopmental variables	602 (77%)
Neurological examination total score	594 (76%)
Early Childhood Behavioral Questionnaire (ECBQ)	592 (76%)
Child Behavioral Checklist (CBCL)	591 (76%)
Quantitative Checklist for Autism in Toddlers (Q-CHAT)	591 (76%)
Cognitively Stimulating Parenting Scale (CSPS)	583 (75%)
Parenting Scale: primary caregivers’ laxness, over reactivity, verbosity	589 (75%)
Parenting Scale: secondary caregivers’ laxness, over reactivity, verbosity	517 (66%)

**TABLE 3:** Tests and completion rates for eye tracking assessments.

Eye-tracking task	*N* (%)
Gap-overlap	602 (77)
Non-social contingency	597 (76)
Visual search	597 (76)
Fishtanks	596 (76)
Cognitive control	585 (75)
Working memory	585 (75)
Emotions	576 (74)
Smooth pursuit fixation	568 (72)
Fixation	484 (64)
Scenes	483 (61)
Static images	481 (61)
Entire eye-tracking battery completed	453 (58)

#### The Bayley Scales of Infant and Toddler Development, Third Edition (Bayley-III)

**FIGURE 9:**
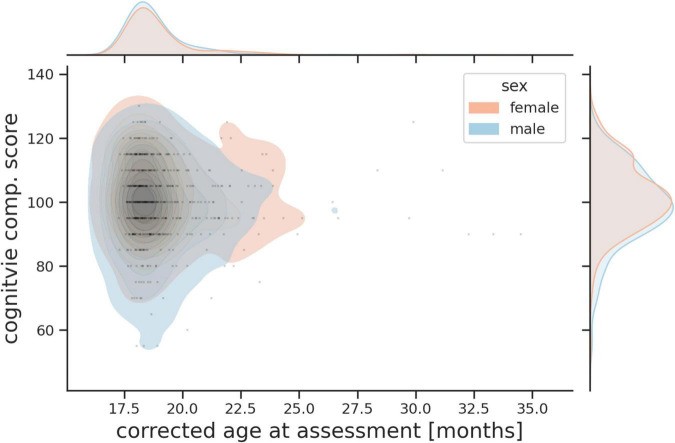
Probability plot showing age of assessment and combined Bayley III cognitive score for boys and girls.

Assessed toddlers’ cognitive, language (receptive and expressive) and motor abilities (gross and fine) using age normed standardized scores (mean = 100, SD = 15) (Albers and Grieve, 2007). The age of assessment and distribution of Bayley III cognitive scores for boys and girls are shown in Figure 9.

#### The Neurological Examination of Infant/Child

Used 26 non-age dependent items to assess cranial nerve function, posture, movements, tone, and reflexes (Haataja et al., 1999).

### Behavioral Questionnaires Completed by Primary Caregivers

#### Early Childhood Behavior Questionnaire

This measures dimensions of temperament, referring to individual differences in reactivity and self-regulation (Putnam and Rothbart, 2006). The Early Childhood Behavior Questionnaire (ECBQ) describes three broad scales: Surgency, characterized by impulsivity, intense pleasure seeking and high activity levels; Negative Affectivity, which refers to the disposition to experience aversive affective states, such as anger, fear, anxiety, shame, and disgust; Effortful Control, which refers to the capacity to inhibit/activate a behavioral response by focusing attention.

#### Child Behavioral Checklist for Ages 1.5–5

Which is a 100-item measure on the frequency of behavioral and emotional problems in young children (Achenbach and Ruffle, 2000). The Child Behavioral Checklist (CBCL) yields scores for seven problem behavior syndrome subscales: Emotionally Reactive, Anxious/Depressed, Somatic Complaints, Withdrawn, Sleep Problems, Attention Problems, and Aggressive Behavior. Scores are also derived for Externalizing Problems, Internalizing Problems, and Total Problems.

#### Quantitative Checklist for Autism in Toddlers

A 25-item questionnaire designed to assess potential autistic traits in children (Allison et al., 2008).

#### Cognitively Stimulating Parenting Scale

Adapted from Wolke et al. (2013), which assesses the availability and variety of experiences that promote cognitive stimulation in the home. This includes availability of educational toys, parental interactions such as teaching words or reading stories, and cognitively stimulating activities such as family excursions. The version of the Cognitively Stimulating Parenting Scale (CSPS) used here was updated to include four items now widely used by toddlers (i.e., iPhone and Apps) (Bonthrone et al., 2021). Scores from the 28 items included in the CSPS can be aggregated to provide an overall cognitively stimulating parenting score.

#### Parenting Scale

Is a 30-item rating scale that measures dysfunctional parenting in discipline situations (Arnold et al., 1993). Parents are asked to indicate their tendency to use specific discipline practices using a 7-point scale. The Parenting Scale identifies three different suboptimal parenting styles, as well as a total score providing a dysfunctional parenting index. Over-reactivity indicates authoritarian and coercive discipline practices; Laxness, in contrast, describes a permissive parent who is inconsistent in providing discipline; Verbosity refers to a parenting style characterized by lengthy and ineffective verbal reprimands. Primary careers were usually mothers and secondary careers usually fathers.

#### Eye-Tracking

Used to obtain data on a number of cognitive processes. The Tobii TX-300 (Tobii AB, Sweden) gaze tracking system was used to record the temporal and spatial features of the children’s direction of gaze in 609 infants (78%) at a median age of 18 months + 12 days (range 17 + 8 − 34 + 15). The battery of tasks comprised a series of animated video clips designed to measure endogenous and exogenous visual attention (Elsabbagh et al., 2009, 2013; Gliga et al., 2009; Wass et al., 2011). Extracted metrics included visual engagement and disengagement, efficiency of attention shifting, social and non-social attention and memory guided choices and visual search. The list of tests and completion rates are shown in Table 3. A manuscript describing the tasks and the results in detail has been submitted for publication (Braithewaite et al., submitted). The project codebook details the variables to be released, while the rich meta-data from these tests may be available through discussion with the dHCP investigators.

### Genomic Data

#### Genetic Data

Saliva samples were collected at the initial neonatal MRI data acquisition and 18-month old infant timepoints using Oragene DNA OG-250 kits (DNAGenotek Inc., Kanata, Canada). The genotyping was performed on only one sample (usually the first). There are no linked maternal or paternal samples. Samples were genotyped on the Illumina Infinium Omni5-4 array v1.2, which comprises a total of 4327108 single-nucleotide polymorphisms (SNPs), by NIHR BioResource Centre Maudsley Genomics & Biomarker Core Facility. Genotyping was undertaken in two batches. Basic quality control was performed by the Department of Biostatistics & Health Informatics, King’s College London for the combined dHCP batches and a small additional independent study cohort. Raw Illumina microarray genotype image (IDAT) files were uploaded into GenomeStudio and processed according to the GenomeStudio quality control Standard Operating Procedure (Patel et al., 2022).^8^ Data was then further processed according to a pipeline which identified and removed samples with call rates below 95% (Patel et al., 2022). It also identified gender mismatches and potential heterozygosity outliers which are flagged in the metadata files. SNP data are available for 731 infants.

#### Methylation Data

Saliva-derived DNA from each sample was treated with sodium bisulfite [Zymo Research EZ-96 DNA Methylation Kit (D5004)]. DNA methylation was quantified using the Illumina Infinium HumanMethylationEPIC BeadChip Kit. Methylation analysis for the dHCP samples was undertaken alongside two additional independent study cohorts. A randomized sample layout was generated using key study parameters including all study cohorts, with Omixer R/Bioconductor package (Sinke et al., 2021).^9^ Saliva samples have been processed for 739 infants, including a subset with samples taken at birth and repeated at the 18-month visit, but QC has yet to be carried out.

## Governance and Access

The study was approved by the United Kingdom Health Research Authority (Research Ethics Committee reference number: 14/LO/1169) and written parental consent was obtained in every case for imaging and open data release of the anonymized data. The main imaging data, essential metadata and the collateral data, will be available after accepting a data sharing agreement. Downloaded data should not be passed on to third parties outside the research group, and no attempt should be made to de-anonymize the data which have been face stripped to prevent attempts at facial recognition.

The preliminary data releases are currently available to download by academic torrent *via* the dHCP website (see text footnote 1). The primary long-term site for curation and access of the full data release will be the National Institute for Mental Health (NIMH) data repository portal at https://nda.nih.gov/edit_collection.html?id=3955.

### Examples of Developing Human Connectome Project Data Use

The preliminary data releases of a proportion of the images have been available to scientists since 2019. There datasets have been accessed frequently and already a large number of studies have been published using dHCP data. These include studies of prenatal opioid exposure (Merhar et al., 2021), cerebral gene expression (Ball et al., 2020), the effects of preterm birth on brain structure and function (Dimitrova et al., 2020; Kline et al., 2020; Eyre et al., 2021), the development of specific cognitive functions (Li et al., 2020), and the neural response to noxious stimuli (Baxter et al., 2019), as well as a number of analyses of brain connectivity and growth (Eyre et al., 2021; Wang et al., 2021; Bethlehem et al., 2022). The data have been widely used to develop novel imaging analytic methods (Ding et al., 2020; Collins-Jones et al., 2021; Grigorescu et al., 2021) and to define new approaches to understanding brain development (Adamson et al., 2020; O’Muircheartaigh et al., 2020).

## Discussion

We describe here the main neonatal data release of the Developing Human Connectome Project which includes 887 datasets from 783 subjects. We are releasing data from all steps in the project, from the initial images through intermediate steps in processing, to results from running our processing pipelines. The aim is to allow researchers to work with the data as they wish, without pre-filtering the available selection. In the majority of cases high quality images across all modalities are available, and are linked to rich collateral data, although practical issues, notably the COVID-19 pandemic, led to some incomplete ascertainment.

Each image acquisition that contributes to the dHCP collection was individually optimized both to take account of the properties of the developing neonatal brain and to achieve the most efficient total examination. After the initial piloting in which the head-foot location of the imaging volume was set, the scanner operated without pause for the entire examination.

Virtually all subjects were examined during natural sleep, so available time for imaging was constrained. We took steps to reduce the risk that infants would awaken by minimizing preparation time after feeding was complete, improving the patient-handling equipment (see Hughes et al., 2017 for details) and modifying the scanner software to avoid sudden changes in acoustic conditions that might create a startle response. Despite these precautions some babies did wake up during the scanning session, but it was often possible to re-settle them and the protocol was designed to allow restart with minimal time penalty, particularly for the dMRI, which was the longest single acquisition. Although precise information about whether a baby woke up during image acquisition was not recorded, it would be of interest in future studies exploring the specific relationship between imaging measures and behavior.

However, even those babies that continued to sleep often moved sufficiently to impair the data quality of the advanced images being collected, so data were motion corrected, either as part of a motion corrected image reconstruction (Anatomical T2w and T1w FSE sequences, but not MPRAGE) or as part of the data processing pipelines, each of which had motion correction steps included. These pipelines were designed and optimized specifically for neonatal data, and software for pipelines is freely available.^10^ Full details are available as part of the data download documentation.

The data from the dHCP naturally sits within a context of other connectome-oriented collections and will be curated alongside many similar resources by the National Institutes of Mental Health in the large multimodal neuroinformatic data repository. The dHCP neonatal data collection will prove valuable to a broad range of users and that it will complement and augment other available materials. Taken together with the dHCP fetal data release, this collection provides what is currently a unique observational resource that captures information on the developing human brain at a key stage of rapid growth and change. The companion genetic and follow-up behavioral resources, as well as atlases, which will be accessed from the same locations, can provide rich materials to address a range of scientific and clinical questions. The data are already being widely used.

## Data Availability Statement

The datasets presented in this study can be found in online repositories. The names of the repository/repositories and accession number(s) can be found below: http://www.developingconnectome.org/data-release/third-data-release/, 1; https://nda.nih.gov/edit_collection.html?id=3955, 3955.

## Ethics Statement

The studies involving human participants were reviewed and approved by United Kingdom Health Research Authority (Research Ethics Committee reference number: 14/LO/1169). Written informed consent to participate in this study was provided by the participants’ legal guardian/next of kin.

## Author Contributions

AE, DR, SMS, and JH obtained funding. SAS, LC-G, AG, EH, JH, SM, RN, AP, RT, J-DT, and JH developed acquisition methods. JAlm, JAll, TA, SA, AC, KC, RC, AD, LD, S-RF, NH, EH, CO’K, HP, JS, NT, KV, and JW collected data. DR, SMS, AA, JAn, TA, MB, LB, JBr, DC, JC, SC, MD, ED, S-RF, SPF, JG, SH, MJ, SJ, VKa, GL, AM, FM, JO’M, JP-P, MP, ER, AS, SS, TT, J-DT, AU, RW, JH, and JBo developed analysis methods. AE, SMS, TA, SA, DB, LB, EB, OC, DC, RD, ED, EJ, VKy, CN, JO’M, MP, MR, LW, and LM analyzed data. AE, DR, TA, DC, HC, KD, S-RF, NH, VKa, MP, AP, and JH prepared manuscript. All authors have reviewed the manuscript.

## Conflict of Interest

The authors declare that the research was conducted in the absence of any commercial or financial relationships that could be construed as a potential conflict of interest.

## Publisher’s Note

All claims expressed in this article are solely those of the authors and do not necessarily represent those of their affiliated organizations, or those of the publisher, the editors and the reviewers. Any product that may be evaluated in this article, or claim that may be made by its manufacturer, is not guaranteed or endorsed by the publisher.

## Footnotes

1
www.developingconnectome.org

2
http://www.developingconnectome.org/study-inclusion-and-exclusion-criteria/

3
https://biomedia.github.io/dHCP-release-notes/

4
www.fmrib.ox.ac.uk/

5
www.mrtrix.org

6
https://brain-development.org/brain-atlases/atlases-from-the-dhcp-project/

7
https://nda.nih.gov/edit_collection.html?id=3955

8
https://khp-informatics.github.io/COPILOT/index.html

9
http://www.bioconductor.org/packages/release/bioc/html/Omixer.html

10
https://biomedia.github.io/dHCP-release-notes/open-resources.html
